# Synergistic Radiosensitization Mediated by Chemodynamic Therapy *via* a Novel Biodegradable Peroxidases Mimicking Nanohybrid

**DOI:** 10.3389/fonc.2022.872502

**Published:** 2022-05-10

**Authors:** Jun Zhang, Dazhen Jiang, Meng Lyu, Shiqi Ren, Yunfeng Zhou, Zhen Cao

**Affiliations:** ^1^ Department of Radiation and Medical Oncology, Hubei Key Laboratory of Tumor Biological Behaviors, Hubei Cancer Clinical Study Center, Zhongnan Hospital of Wuhan University, Wuhan, China; ^2^ Key Laboratory of Artificial Micro- and Nano-Structures of Ministry of Education, School of Physics and Technology, Wuhan University, Wuhan, China; ^3^ BGI College & Henan Institute of Medical and Pharmaceutical Sciences, Zhengzhou University, Zhengzhou, China

**Keywords:** mesoporous silica, peroxidase mimetic, reactive oxygen species, radiotherapy, chemodynamic therapy

## Abstract

**Purpose:**

Reactive oxygen species (ROS) are practically essential in radiotherapy to damage cancer cells; however, they are always inadequate for some malignant entities. Here, we designed a biodegradable mesoporous silica decorated with hemin and glucose oxidase (GOD@Hemin-MSN) to generate a chemodynamic therapy in order to enhance the killing capacity of radiotherapy.

**Methods:**

Mesoporous silica, as an outstanding drug carrier, can deliver hemin and glucose oxidase to the tumor site. With high level of metabolism activity, cancer cells are abundant in glucose, which can be oxidized into H_2_O_2_ by glucose oxidase (GOD) on site. The generated H_2_O_2_ is subsequently converted into intracellular ROS, especially hydroxyl radical within the tumor microenvironment, by hemin, which has mimetic peroxidase properties. By this means, the ROS can be supplemented or enriched to facilitate the killing of tumor cells.

**Results:**

The chemodynamic therapy induced by GOD@Hemin-MSN produced quantities of ROS, which compensated for their inadequacy as a result of radiotherapy, and exhibited remarkable antitumor efficacy, with a tumor inhibition rate of 91.5% in A549 tumor-bearing mice.

**Conclusion:**

This work has validated GOD@Hemin-MSN as a radiosensitizer in chemodynamic therapy, which showed biocompatibility and potential for translational application.

## Introduction

Compared with conventional enzymes, nanozymes with some enzyme-mimicking properties have exhibited multiple applications in clinical diagnosis (1–4), bioanalysis (4, 5), biosensors (6, 7), and disease treatment (8, 9), with advantages of facile synthesis, tunable catalytic activities, and high stability. Radicals including $O_{2}{}^{-}$ , $O_{2}{}^{2-}$ , OH·, and OOH·, known as reactive oxygen species (ROS), are essentially involved in many processes of cellular metabolism, and some nanozymes are capable of regulating the level of intracellular ROS, which provides possibilities for mediating ROS generation to achieve desired therapeutic outcomes in clinical practice (10, 11). The tumor microenvironment (TME) has unique characteristics including mild acidity (6, 12, 13) and an overexpressed H_2_O_2_ (14–16), and a highly complex TME might impair the catalytic effects of nanozymes, resulting in failure of obtaining the desired therapeutic outcomes (17, 18). On the other hand, such features could be utilized by some H_2_O_2_-responsive nanozymes to realize specific antitumor therapies. These nanozymes should be able to decompose H_2_O_2_ into ROS due to their peroxidase (POD)-mimicking properties under specific TME conditions, i.e., mildly acidic (19–21). However, the amount of intracellular H_2_O_2_ in tumor cells is still insufficient to generate enough ROS for achieving the desired therapeutic outcomes (22). Hence, a good way to treat tumors is to increase the level of intracellular H_2_O_2_ and convert it into sufficient ROS in tumor cells.

As one of the most widely used and effective treatments for local control of malignant tumors, radiotherapy (RT) still needs to be improved in many ways, especially for radiation-insensitive tumors (23, 24). Radiotherapy causes tumor cells to undergo apoptosis *via* the induction of direct and indirect damage on the DNA, with the indirect damage primarily caused by the ROS produced during the process of RT (25). Limited by the tolerable dose of adjacent normal tissues, the delivery of radiation with sufficient high dose to specific tumor sites is not always achievable, which seriously impairs the treatment efficacy of RT, especially for radiation-insensitive tumors (26–28). Improving the treatment efficacy of malignant tumors using RT still remains a prominent problem in clinical practice (29), and increasing the energy deposit of ionizing radiation in the tumor region should be a good solution (30). To this end, nano-radiosensitizers with remarkable physical and chemical properties to enhance the killing effect of radiation on tumor cells have caught the attention of radiation oncologists in recent years (22, 24), providing significant opportunities for tumor RT sensitization. Moreover, radiosensitizers with specific targeting abilities can aggregate inside tumor cells with minimal adverse effects on normal tissue cells. Some nano-radiosensitizers enhance the energy deposition on tumor tissues by taking advantage of high-*Z* elements, which improves the photoelectric effect of radiation rays (31–34). Hypoxia, as an obvious characteristic of the TME, hinders the treatment efficacy of RT, resulting in radioresistance, a category of radiosensitizers designed to focus on producing stable radicals in the TME to damage DNA (35). For example, the overexpression of H_2_O_2_ in the TME could be catalyzed to oxygen by nanozyme radiosensitizers such as MnO_2_ (33, 36) and Pt-based nanomaterials (Yan 31, 37), among others. Besides, RT is usually combined with other therapeutic methods such as photothermal therapy (38, 39), photodynamic therapy (40, 41), and chemotherapy (42, 43) to obtain synergistic therapeutic outcomes. These radiosensitizers contribute directly or indirectly to ROS generation to induce accumulated tumor cell apoptosis, enhancing RT performance but maintaining a lower toxicity.

**Scheme 1:**
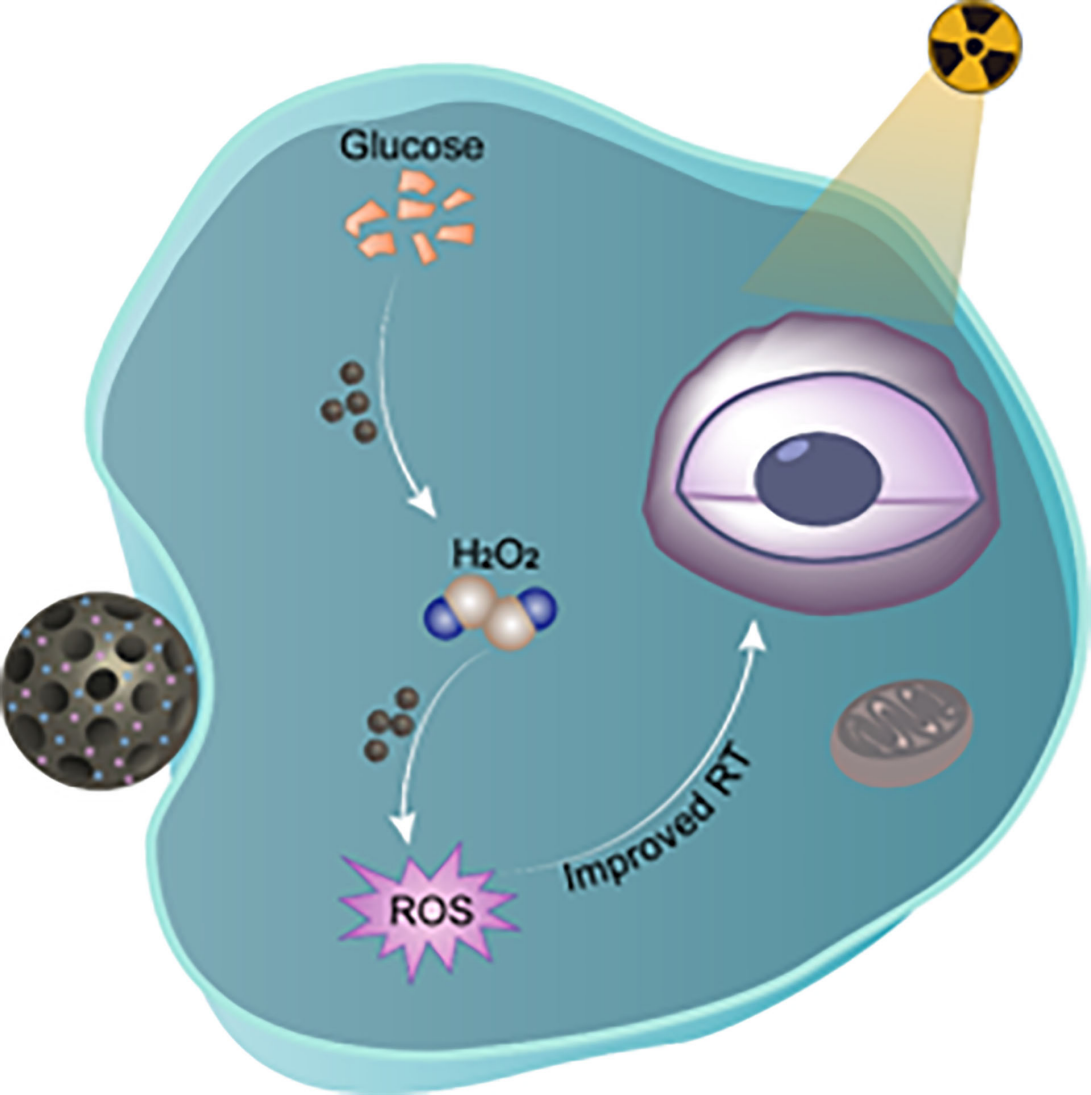
Illustration of biodegradable peroxidases mimicking nanohybrid GOD@Hemin-MSN for chemodynamic therapy to realize radiosensitization. Zhang et al. Radiosensitization Frontiers in

ROS can induce indirect tumor cell apoptosis; however, they can also be eliminated by tumor cells themselves, thereby resulting in a tumor’s radioresistance (44, 45). Therefore, incremental amounts of ROS are required to overcome the radiation tolerance induced by the self-protective mechanisms of tumor cells. Here, we designed a biodegradable nanohybrid for radiosensitization by immobilizing glucose oxidize (GOD) and hemin on the surface of mesoporous silica nanospheres (MSN) to induce more production of ROS, as shown in **Scheme 1**. Initially, the nanohybrid was selectively delivered to the tumor site *via* an enhanced permeability and retention effect in the TME. Then, the GOD on the nanohybrid catalyzes intracellular glucose into sufficient H_2_O_2_, which is decomposed to generate incremental amounts of ROS due to the intrinsic POD enzyme-mimicking activity of hemin in order to achieve chemodynamic therapy. The carrier MSN is biodegradable *in vivo* and can be metabolized in the human body to ensure biological safety. By this means, incremental production of intracellular ROS facilitates the killing effect of ionizing radiation, with minimal damage to adjacent normal tissues.

## Results and Discussion

**Figure 1:**
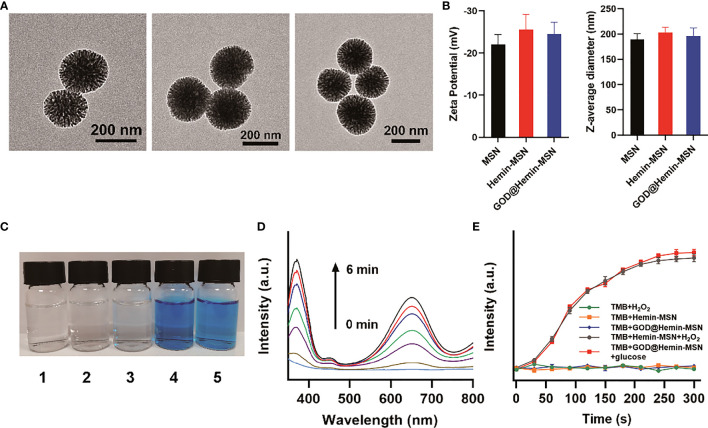
Characterization. **(A)** TEM images of mesoporous silica nanospheres (MSN), hemin-MSN, and MSN decorated with hemin and glucose oxidase (GOD@Hemin-MSN) (*from left to right*). **(B)** Zeta potential and *Z*-average diameter measurements of MSN, hemin-MSN, and GOD@Hemin-MSN. **(C)** Images of the reaction of 3,3′,5,5′-tetramethylbenzidine (TMB) solution (0.1 mM) to (*1*) H_2_O_2_, (*2*) hemin-MSN, (*3*) GOD@Hemin-MSN, (*4*) hemin-MSN+H_2_O_2_, and (*5*) GOD@Hemin-MSN+glucose. **(D)** UV–vis spectra of the reaction of TMB solution to GOD@Hemin-MSN+glucose with time variation. **(E)** Time-dependent changes of the absorbance of TMB solution at 652 nm with various solutions.

The design of the present nanohybrid includes decorating GOD and hemin on the MSN. **Figure 1A** demonstrates spherical MSN, Hemin-MSN, and GOD@Hemin-MSN. Moreover, GOD@Hemin-MSN is uniform, with an average size of approximately 190 nm according to particle size statistics (**Supplementary Figure S1**), which is consistent with the *Z*-average diameter (**Figure 1B**). In addition, no obvious changes in the zeta potential or *Z*-average diameter were observed after hemin or GOD, and hemin was immobilized on the MSN. The X-ray photoelectron spectroscopy (XPS) measurements of the Si2p orbit of GOD@Hemin-MSN in **Supplementary Figure S2** demonstrate a binding energy of 103.6 eV, confirming the existence of Si^4+^.

Subsequently, the POD-like property of GOD@Hemin-MSN was then evaluated using 3,3′,5,5′-tetramethylbenzidine (TMB) substrate. TMB is transparent and colorless, but turns blue in the presence of both H_2_O_2_ and peroxidase. Comparative analyses were performed in TMB solutions with various combinations, including H_2_O_2_, hemin-MSN, GOD@Hemin-MSN, hemin-MSN+H_2_O_2_, and GOD@Hemin-MSN+glucose, as shown in **Figure 1C**. No obvious change in color was observed in TMB in the presence of H_2_O_2_, hemin-MSN, or GOD@Hemin-MSN, proving that they could not oxidize TMB alone. However, the TMB solution turned blue when both hemin-MSN and H_2_O_2_ were added, suggesting that TMB was oxidized. Similar changes could be found in the presence of GOD@Hemin-MSN and glucose. It should be noted that H_2_O_2_ was not added in group 5, where the oxidization of TMB occurred induced by catalyzing glucose to H_2_O_2_ using GOD@Hemin-MSN. Moreover, time-dependent changes in the absorbance spectra of the TMB solution in the presence of GOD@Hemin-MSN and glucose were demonstrated, shown in **Figure 1D**. The characteristic peaks of the absorbance spectra at 370 and 652 nm increased over time. Comparison experiments of TMB with various solutions are demonstrated in **Figure 1E**. Consistent with the results shown in **Figure 1C**, negligible changes were observed regarding the absorbance intensity at 652 nm in the H_2_O_2_, hemin-MSN, or the GOD@Hemin-MSN group. However, both hemin-MSN+H_2_O_2_ and GOD@Hemin-MSN+glucose exhibited remarkable catalytic activity with time variance. Moreover, we also studied the effects of temperature and pH on the POD activity of GOD@Hemin-MSN+glucose. The results are shown in **Supplementary Figure S3**. It can be inferred that 37°C was a suitable temperature for POD activity; however, the group in 50°C exhibited more obvious effects. Meanwhile, the POD activity was higher in the mildly acidic solution. These results confirmed the significant enzyme-like properties of GOD@Hemin-MSN in the conversion of glucose into ROS.

**Figure 2:**
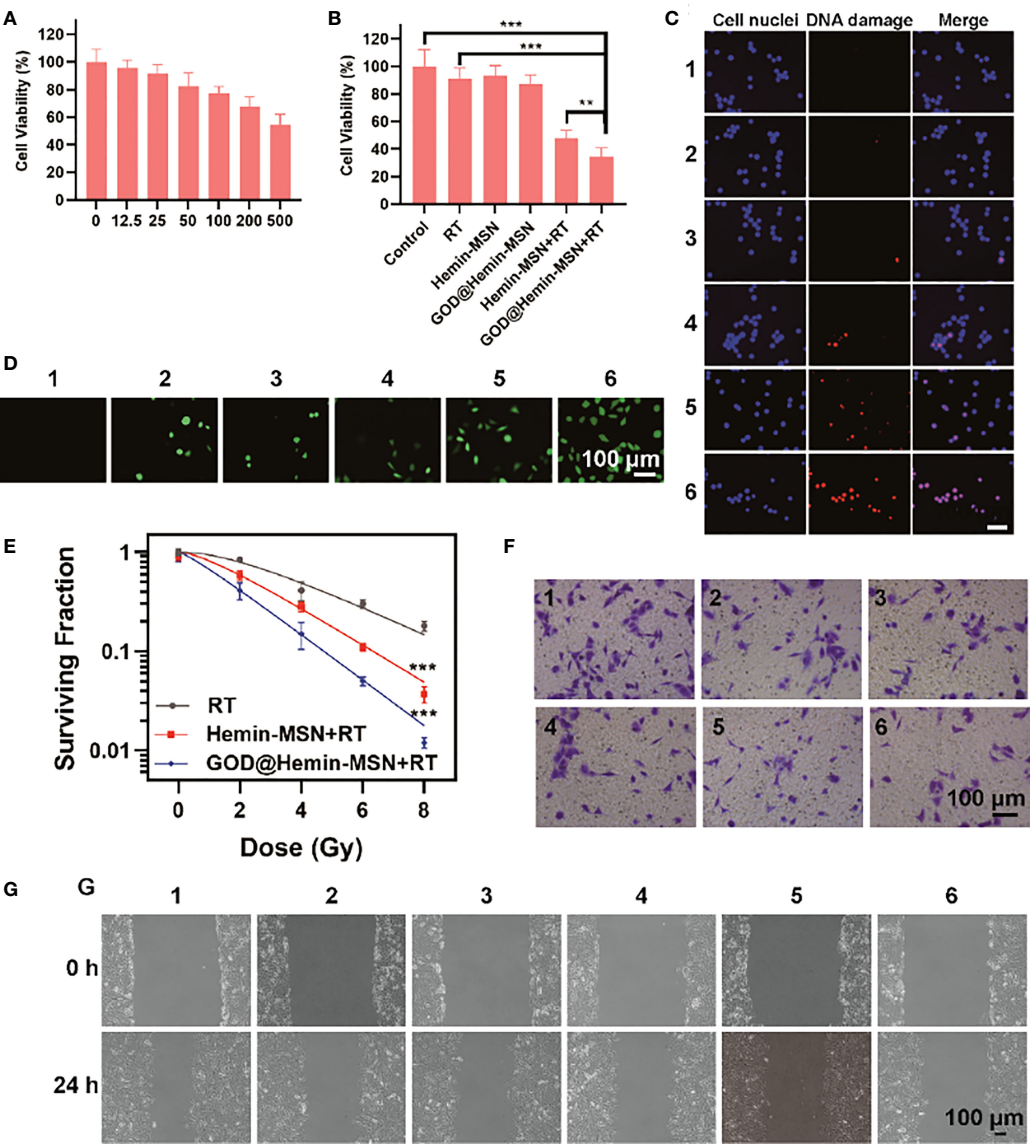
Cell experiments. **(A)** Cell viability of normal human lung fibroblast (NHLF) cells after incubation with mesoporous silica decorated with hemin and glucose oxidase (GOD@Hemin-MSN) at various concentrations. **(B)** Cell viability of A549 cells after various treatments. **(C)** Confocal laser scanning microscopy (CLSM) images of γ-H_2_AX staining. **(D)** CLSM images of reactive oxygen species (ROS) staining. **(E)** Colony formation. **(F)** Transwell experiments after various treatments. **(G)** Wounding assay. *1*, control; *2*, radiotherapy (RT); *3*, hemin-MSN; *4*, GOD@Hemin-MSN; *5*, hemin-MSN+RT; and *6*, GOD@Hemin-MSN+RT. Data are presented as the mean ± SD using one-way ANOVA with Tukey’s multiple comparison tests. ***p < 0.001; **p < 0.01.

Cytotoxicity analysis was performed on a normal human lung fibroblast (NHLF) cell line using the CCK-8 assay. To assess the cytotoxicity of GOD@Hemin-MSN, we incubated NHLF cells with GOD@Hemin-MSN at various therapeutic concentrations (12.5, 25, 50, 100, 200, and 500 μg/ml); the group without any treatment was set as the control group. As shown in **Figure 2A**, the cell survival rate decreased slightly with the increase of GOD@Hemin-MSN concentration. It is notable that the cell survival rate still reached more than 60% even at the highest concentration (500 μg/ml), indicating that GOD@Hemin-MSN exhibits no obvious cytotoxicity and good biocompatibility. Subsequently, to verify its killing effect on tumor cells (**Figure 2B**), A549 cells, human lung adenocarcinoma cells, were treated with 6 different treatment combinations: 1) control, 2) RT, 3) hemin-MSN, 4) GOD@Hemin-MSN, 5) hemin-MSN+RT, and 6) GOD@Hemin-MSN+RT. The RT dose was 6 Gy, and the equivalent concentration of hemin-MSN was 50 μg/ml. No obvious cytotoxicity was observed in the control, RT, and hemin-MSN treatment groups. In the hemin-MSN+RT and GOD@Hemin-MSN+RT treatment groups, however, apparent cell death was observed, while the GOD@Hemin-MSN+RT treatment group exhibited much more obvious cytotoxicity than did the hemin-MSN+RT treatment group. The confocal laser scanning microscopy (CLSM) images of the live/dead staining assay further confirmed these results (**Supplementary Figure S4**). These results indicate that GOD is capable of oxidizing glucose to produce abundant amounts of H_2_O_2_, which, when combined with endogenous H_2_O_2_, was catalyzed by hemin to produce incremental amounts of ROS, realizing chemodynamic therapy for a greater killing effect on tumor cells in addition to standard RT. We also noticed, after 6 Gy radiotherapy, that the cell killing rate of the GOD@Hemin-MSN+RT group was significantly higher than that of RT alone, indicating that GOD@Hemin-MSN plays a synergistic role with RT and improves the therapeutic effects. DNA double-strand break (DSB) is the major form of lesion caused by RT that induces the apoptosis of tumor cells. Gamma-H_2_AX (γ-H_2_AX), a well-known sensitive DNA damage molecular marker, was used for fluorescence staining to indicate DSBs. As shown in **Figure 2C**, γ-H_2_AX foci (red fluorescence) were observed in the cell nuclei, and remarkable amounts of DNA damage were found in the GOD@Hemin-MSN+RT group. Corresponding quantitative analysis was conducted using ImageJ software. It is worth noting that hemin-MSN with RT caused 34.4% of the γ-H_2_AX foci; however, GOD@Hemin-MSN combined with RT caused 78.2% of the γ-H_2_AX foci, indicating the impressive radiosensitization ability of the nanohybrid (**Supplementary Figure S5**). In order to confirm and clarify whether ROS were really produced incrementally, the cells were incubated with dichlorodihydrofluorescein diacetate (DCFH-DA) to examine the ROS production in the different groups (**Figure 2D**). The GOD@Hemin-MSN group showed green fluorescence, confirming that GOD@Hemin-MSN can consume glucose to produce H_2_O_2_ and, further, to generate ROS. The GOD@Hemin-MSN+RT group showed stronger fluorescence, indicating a higher ROS production. In other words, the GOD@Hemin-MSN nanohybrid can be combined with RT to improve the therapeutic effects by producing incremental amounts of ROS. The results of colony formation are shown in **Figure 2E**. A marked inhibition of the proliferation of tumor cells was observed in the GOD@Hemin-MSN+RT group. The sensitization enhancement ratio of GOD@Hemin-MSN was calculated as 1.60, which was notably higher than that of hemin-MSN (1.32). All these results provided proof that chemodynamic therapy mediated by GOD@Hemin-MSN can substantially improve the killing efficiency of RT through more ROS production. Cell invasion occurs in the process of metastasis of malignant cells. Therefore, studying the mechanisms involved has important implications on a variety of physiological/pathological processes. The invasion and scratch experiments (**Figures 2F, G**) also showed similar results, in which the GOD@Hemin-MSN+RT group had the widest gap among all groups, indicating the strongest suppression on the invasion and the lowest cell fusion rate of A549 cells. With these data, it was deduced that GOD@Hemin-MSN can inhibit A549 cell metastasis by affecting its adhesion, invasion, and migration.

**Figure 3:**
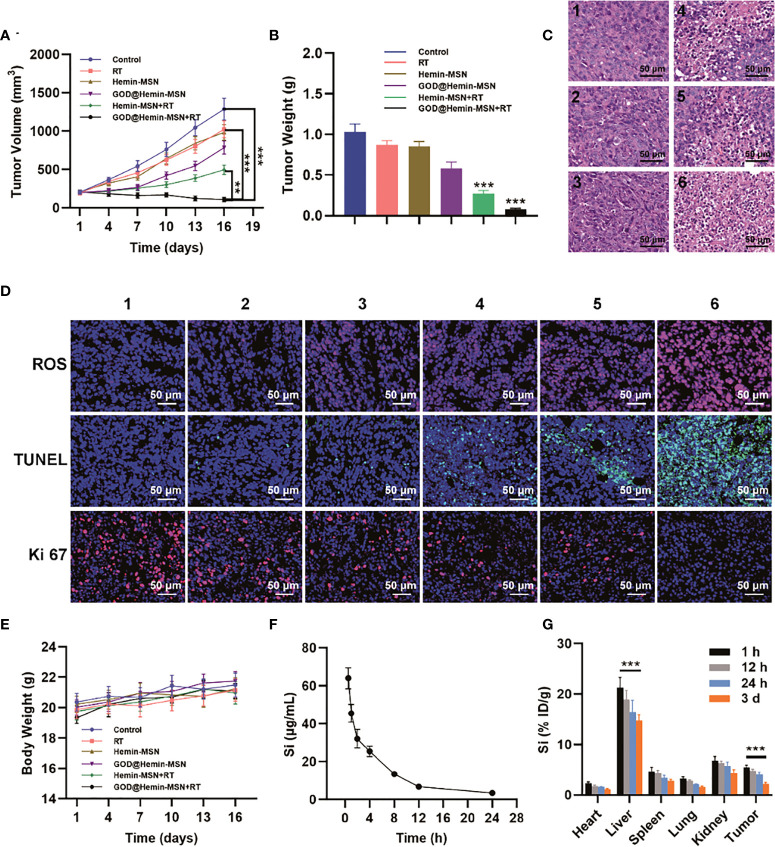
Radiosensitization *in vivo* in tumor xenograft mice. **(A)** Tumor volume changes. **(B)** Tumor weights. **(C)** Hematoxylin–eosin (H&E) staining of the tumor slices [group *1*, control; group *2*, radiotherapy (RT); group *3*, hemin-MSN; group *4*, GOD@Hemin-MSN; group *5*, hemin-MSN+RT; and group *6*, GOD@Hemin-MSN+RT]. **(D)** Immunofluorescence staining. **(E)** Body weights in each group. **(F)** Pharmacokinetic curves. **(G)** Biodistribution at 1, 12, and 24 h and at 3 days post-injection of GOD@Hemin-MSN [the same groups as in **(C)**]. Data are presented as the mean ± SD using one-way ANOVA with Tukey’s multiple comparison tests. ***p < 0.001; **p < 0.01.

On account of the promising *in vitro* data, the *in vivo* antitumor efficacy of GOD@Hemin-MSN combined with RT was assessed on A549 tumor xenograft mice. When the tumor volume reached approximately 200 mm^3^, the mice were divided equally into 6 groups and subjected to various treatments, as follows: 1) control [200 µl phosphates-buffered saline (PBS)]; 2) RT (6 Gy); 3) hemin-MSN (50 μg/ml); 4) GOD@Hemin-MSN (50 μg/ml); 5) hemin-MSN (50 μg/ml) + RT (6 Gy); and 6) GOD@Hemin-MSN (50 μg/ml) + RT (6 Gy). The day the mice were first treated was recorded as day 1. Then, every third day, the body weights and tumor volumes of the mice were monitored and recorded. All mice in the 6 groups were euthanized on day 16. As demonstrated in **Figures 3A, B** and **Supplementary Figure S6**, the tumor volume in the control group increased significantly, reaching an average of 6.3 times on day 16 compared with that on day 1. Most of the mice in the GOD@Hemin-MSN+RT group survived. The tumor volumes in the RT, hemin-MSN, and GOD@Hemin-MSN groups were moderately suppressed. Notably, mice treated with hemin-MSN+RT exhibited remarkably reduced tumor volumes, which was consistent with the results of the *in vivo* experiment, indicating that hemin-MSN imposed a synergistic killing effect when combined with RT on the tumor region; that is, it sensitized RT by producing incremental amounts of ROS. However, this synergistic effect was still restrained by the limited H_2_O_2_ in tumor tissues. Therefore, GOD@Hemin-MSN compensated for the shortage of H_2_O_2_ in the TME and exhibited a much higher synergistic damage to the tumor in mice administered with GOD@Hemin-MSN followed by RT, achieving tumor inhibition rates as high as 91.5%. Hematoxylin–eosin (H&E) staining (**Figure 3C**) of the tumor tissue sections of each group also further confirmed our conclusion, as it could be seen that there were significant and abundant areas of necrosis and nuclear pyknosis in the tumor tissues of the GOD@Hemin-MSN+RT group. As shown in **Figure 3D**, the strongest green fluorescence was observed in tumor specimens of the GOD@Hemin-MSN+RT group. The underlying mechanism can be explained as follows: GOD on the GOD@Hemin-MSN nanohybrid catalyzed the intracellular glucose to produce abundant H_2_O_2_, which was subsequently catalyzed by hemin to produce incremental amounts of ROS. On the other hand, with the presence of GOD@Hemin-MSN, the ROS produced by ionizing radiation can also be further enhanced, leading to a spike in the ROS levels in the tumor region. Ki-67 and terminal deoxynucleotidyl transferase dUTP nick-end labeling (TUNEL) assays of the tumor tissue specimens of the GOD@Hemin-MSN+RT group further confirmed the enhanced killing efficiency, as indicated by the remarkable proliferation inhibition and tumor cell apoptosis *in vivo.* All treatment methods exhibited no obvious influence on the body weight of mice in all groups within 16 days, as shown in **Figure 3E**. The pharmacokinetic curves in **Figure 3F** demonstrated a blood circulation half-life of GOD@Hemin-MSN of approximately 2.1 h. However, the concentration of Si dropped to 3.5 μg/ml at 24 h post-injection, indicating that GOD@Hemin-MSN was metabolized quickly. The major accumulation of GOD@Hemin-MSN was observed in the kidney and liver at 24 h post-injection, revealing the renal and liver clearance pathway of GOD@Hemin-MSN **(Figure 3G)**. With this method, GOD@Hemin-MSN could be a promising radiosensitizer administered with standard precise intensity-modulated radiotherapy (IMRT) to improve treatment effects or overcome radioresistant tumors for better therapeutic clinical outcomes, such as for the treatment of cancers impossible to be cured by standard RT, although this still needs to be validated in clinical trials.

**Figure 4:**
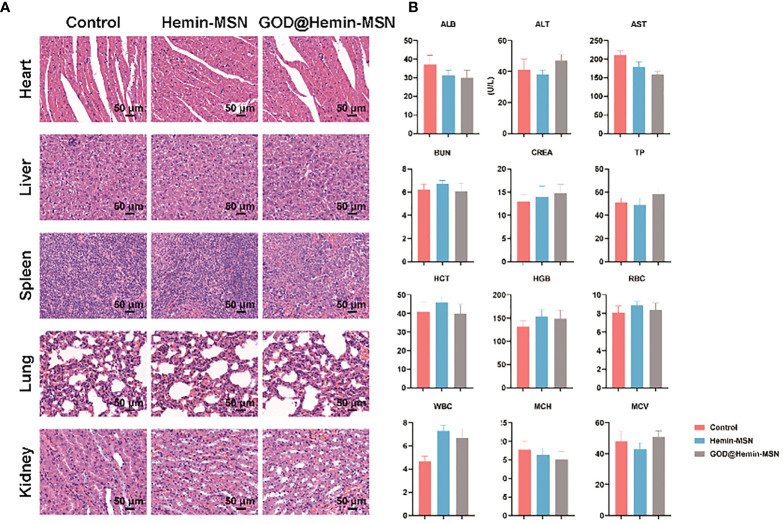
**(A)** Light views of the main organs (heart, liver, spleen, lung, and kidney) with hematoxylin–eosin (H&E) staining. **(B)** Detection of the blood biochemical indicators. ALB, Albumin; ALT, Alanine aminotransferase; AST, Aspartate aminotransferase; BUN, Blood urea nitrogen; CREA, Creatinine; TP, Total protein; HCT, Hematocrit; HGB, Hemoglobin; RBC, Red blood cell; WBC, White blood cell; MCH, Mean corpuscular hemoglobin; MCV, Mean corpuscular volume.

The biocompatibility of the GOD@Hemin-MSN nanohybrid *in vivo* will be the major concern before it becomes clinically practicable. The main organs including the heart, liver, spleen, lung, and kidney of mice administered PBS, hemin-MSN, and GOD@Hemin-MSN were collected for observation with H&E staining (**Figure 4A**). There was no significant variance in the morphology of all the main organs in the three groups observed, indicating the satisfactory histocompatibility of hemin-MSN and GOD@Hemin-MSN. Peripheral blood was also collected from each group for routine blood biochemical test. As shown in **Figure 4B**, no significant difference was found for all blood indicators among the three groups. The results indicate that the GOD@Hemin-MSN nanohybrid and its synergistic RT sensitization strategy are highly biocompatible.

## Conclusion

The radioresistance of tumors can sometimes account for the self-clearance of ROS by the tumor cells themselves, which hinders the therapeutic efficacy of RT clinically. POD-like enzymes can catalyze overexpressed H_2_O_2_ in the TME, which, however, is always insufficient to produce enough ROS to damage the target DNA. We successfully synthesized a novel nanohybrid, GOD@Hemin-MSN, with proven biosafety and biodegradability. GOD@Hemin-MSN can be delivered inside tumor cells and catalyze oxidized glucose to produce abundant H_2_O_2_. The H_2_O_2_ is then converted into sufficient ROS to induce chemodynamic therapy, which synergistically works with the direct and indirect DNA damage of RT to perform the killing of tumor cells more efficiently. Our data from both *in vitro* and *in vivo* experiments suggest that GOD@Hemin-MSN overcomes the shortcomings of a standard RT and could be a promising radiosensitization strategy in clinical practice to reverse radioresistance or provide a more efficient therapy for cancer, but with minimal damage to adjacent normal tissues.

## Data Availability Statement

The original contributions presented in the study are included in the article/**Supplementary Material**. Further inquiries can be directed to the corresponding author.

## Ethics Statement

All experiments complied with the ethical standards in the Declaration of Helsinki and approved by the Ethics Committee of Zhongnan Hospital of Wuhan University. The animal experiment was permitted by the Institutional Animal Care and Use Committee of Wuhan University. The animal study was reviewed and approved by the Ethics Committee of Zhongnan Hospital of Wuhan University.

## Author Contributions

JZ and DJ made substantial contributions to the study concept and design, data analysis and interpretation, and the first draft of manuscript. ZC participated in study design and data analysis, drafting and revising the manuscript, had full access to all of the data in the study and take responsibility for the integrity and the accuracy of the data. ML made substantial contributions to the preparation of experimental materials and discussion of experimental puzzles. SR made substantial contributions to assembling of nanohybrid. YZ made substantial revisions to the manuscript. All authors contributed to the article and approved the submitted version.

## Conflict of Interest

The authors declare that the research was conducted in the absence of any commercial or financial relationships that could be construed as a potential conflict of interest.

## Publisher’s Note

All claims expressed in this article are solely those of the authors and do not necessarily represent those of their affiliated organizations, or those of the publisher, the editors and the reviewers. Any product that may be evaluated in this article, or claim that may be made by its manufacturer, is not guaranteed or endorsed by the publisher.
