# Supplementary material for: Synergistic Radiosensitization Mediated by Chemodynamic Therapy via a Novel Biodegradable Peroxidases Mimicking Nanohybrid
Source: Front Oncol. 2022 May 10;12:872502. doi: 10.3389/fonc.2022.872502 (PMC9128550; doi:10.3389/fonc.2022.872502)
Supplement: Supplementary file 1 [file DataSheet_1.docx]

**Synergistic radiosensitization mediated by chemodynamic therapy via a novel biodegradable peroxidases mimicking nanohybrid**

Jun Zhang^1^, Dazhen Jiang^1^, Meng Lyu^2^, Shiqi Ren^3^, Yunfeng Zhou^1^, Zhen Cao^1^*

^1^ Department of Radiation and Medical Oncology, Hubei Key Laboratory of Tumor Biological Behaviors, Hubei Cancer Clinical Study Center, Zhongnan Hospital of Wuhan University, Wuhan, China, 430071

^2^ Key Laboratory of Artificial Micro- and Nano-Structures of Ministry of Education, School of Physics and Technology, Wuhan University, Wuhan, 430072, China

^3^ BGI College & Henan Institute of Medical and Pharmaceutical Sciences, Zhengzhou University

*Corresponding author: Zhen Cao, Department of Radiation and Medical Oncology, Zhongnan Hospital of Wuhan University, Wuhan, Hubei, PRC 430071, Telephone: +86-159-2631-5546, Email: zhencao@whu.edu.cn.

**Experimental Section**

**Chemicals:** Cetyltrimethylammonium chloride (CTAC) solution (25 wt. % in H_2_O), tetraethyl orthosilicate (TEOS), bis[3-(triethoxysilyl) propyl] tetrasulfide (BTES), hemin, ammonium hydroxide, triethanolamine (TEA), NHydroxysuccinimide (NHS), 1-(3-dimethylaminopropyl)-3-ethylcarbodiimide hydrochloride (EDC), (3-Aminopropyl) triethoxysilane (APTES), Glucose oxidase (GOD), N, N-Dimethylformamide (DMF), were purchased from Sigma-Aldrich.

**Synthesis of Hemin-Silane Precursor:** Hemin (50 mg), EDC (32.78 mg), and NHS (19.68 mg) were dissolved in 5 mL of DMF solution followed by addition of 20 μL of APTES. The system was stirred overnight at room temperature to obtain 5 mL of DMF solution of Hemin-silane precursor (10 mg/mL).

**Synthesis of Hemin-MSN:** CTAC (2 g) and TEA (0.1 g) were dissolved in 20 mL of ultrapure water and stirred for 0.5 h. The system was placed in 65 °C oil base and then added with 1 mL of TEOS. After 1 h of reaction, the mixture of 1 mL of BTES and 0.5 mL of TEOS with 1 mL of the above DMF solution of Hemin-silane precursor was added to the system for another 4 h of reaction under nitrogen atmosphere. The resultant Hemin-MSN products were collected by centrifugation and washed several times with ethanol. After extraction of CTAC in methanol with NaCl, the CTAC-free Hemin-MSN nanoparticles were dissolved in 10 mL of ultrapure water.

**Synthesis of GOx@Hemin-MSN:** The above as-synthesized Hemin-MSN and GOx were added to 30 mL of ultrapure water and stirring for 24 h. The resultant GOx@Hemin-MSN products were collected by centrifugation and then washed with water.

**Physical Characterization of GOD@Hemin-MSN:** The morphology of GOD@Hemin-MSN was observed by transmission electron microscopy (TEM; Tecnai G2 F20 S-Twin, FEI, USA) at 100 keV acceleration voltage. The zeta potential and zeta diameter of the GOD@Hemin-MSN in 1 × PBS in suspension were measured by dynamic light scattering (DLS, Nano-Zen 3600, Malvern Instruments, UK). The surface chemical elements were analyzed by XPS (ESCA-Lab250XI, Thermo Fisher Ltd., USA). The optical absorbance of NPs in the wavelength range of 300-800nm was measured effectively by UV-Vis near-infrared spectrophotometer (CARY5000, Varian Ltd., USA). The phase structures were acquired by means of X-ray diffraction (XRD; Bruker D8 Advance, Germany) with Cu K𝛼 radiation (𝜆 = 0.15406 nm).

**Peroxidase Activity Measurements:** The catalytic experiments and the comparative experiments (monitored by measuring the absorbance at 652 nm) were carried out in phosphate buffer solution (PBS, pH 5, 3 mL) containing TMB (0.1 mM) in the presence of H_2_O_2_ (13 mM), Hemin-MSN, GOD@Hemin-MSN, Hemin-MSN+H_2_O_2_ (13 mM), GOD@Hemin-MSN +glucose (100 μg/mL). The time-dependent absorbance spectra were obtained in PBS containing TMB with GOD@Hemin-MSN +glucose (100 μg/mL). Hemin-MSN and GOD@Hemin-MSN were of equivalent concentration of MSN at 50 μg/mL.

**Cell culture:** The A549 cells (Human non-small cell lung cancer cells, the Cell Bank of the Chinese Academy of Sciences) and normal lung fibroblast NHLF cell line (purchased from the Cell Bank of the Chinese Academy of Sciences) were incubated in RPMI-1640 medium containing 10% FBS in a humidified atmosphere at 37 °C with 5 % CO_2_.

**Cytotoxicity assay (CCK-8):** CCK-8 kit was used to evaluate the dark toxicity of GOD@Hemin-MSN concentration on cells and the effect of different treatment groups on cell viability. Firstly, A549 cells were seeded into 96-well plates at a density of 5×10^3^ cells per well and divided into 7 groups (5 cells per group) for incubation for 24h. Then, the untreated group was used as the control group, and the other 6 groups were incubated with GOD@Hemin-MSN at different concentrations (12.5, 25, 50, 100, 200, 500 μg/mL) for 24 h, respectively. then 10 μL CCK-8 reagent was added to each well. After incubation for 2 h, the absorbance value at the characteristic peak at 450 nm was measured by a microplate analyzer (Rayto-6000 system, Rayto, China). Secondly, A549 cells seeded into 96-well plates at a density of 5×10^3^ cells per well and were divided into 6 groups (5 wells per group): (1) Control, (2) RT, (3) Hemin-MSN, (4) GOD@Hemin-MSN, (5) Hemin-MSN+RT, (6) GOD@Hemin-MSN+RT. Among them, the radiotherapy dose was 6Gy, and the equivalent concentration of MSN was 100μg/mL. The following operations are the same as described in step 1.

**Fluorescence analysis of intracellular ROS:** A549 cells were incubated in a six-well plate at 37℃ for 24 h and divided into 6 groups: (1) Control, (2) RT, (3) Hemin-MSN, (4) GOD@Hemin-MSN, (5) Hemin-MSN+RT, (6) GOD@Hemin-MSN+RT. Among them, the radiotherapy dose was 6Gy, and the equivalent concentration of MSN was 100μg/mL. After corresponding experimental operation, the cells were incubated at 37℃ for 4h. The old culture medium was removed, diluted DCFH-DA was added to the six-well plate, and the cells were cultured at 37℃ for 20 min. The cells were washed with serum-free culture medium for three times, and then the ROS fluorescence level was directly observed using a Japanese laser confocal microscope.

**Colony formation assay:** A549 cells were seeded into 6-well plates with 500 per well and incubated at 37 °C for 24 h. The radiation dose curve experiments were designed into two groups with two complex wells in each group: (1) Hemin-MSN+RT (with an equivalent MSN dose of 100 μg/mL); (2) GOD@Hemin-MSN+RT (with an equivalent MSN dose of 100 μg/mL). After the incubation time reached 2 h, three washings were performed. Five six-well plates were set up for each treatment, and different radiotherapy doses (0, 2, 4, 6, 8 Gy) were performed respectively. 10 days later, cells were stained with Crystal violet dye. This process calls the standard linearquadratic model, counts the colonies covering at least 50 cells, and completes the effective calculation of the colony formation rate. Then, the survival rate of the colony was calculated to realize the evaluation of the effects of various treatments. The process is repeated three times.

**Transwell:** The cell density was adjusted to 1×10^5^ cells /mL using a blood counting plate, 500 μL complete medium containing 20% FBS was added into the lower chamber of Transwell, 200 μL cell suspension was inoculated into the upper chamber of Transwell in an incubator containing 5% CO_2_ at 37℃ for 24h, and the cells were divided into six groups: (1) Control, (2) RT, (3) Hemin-MSN, (4) GOD@Hemin-MSN, (5) Hemin-MSN+RT, (6) GOD@Hemin-MSN+RT. Among them, the radiotherapy dose was 6Gy, and the equivalent concentration of MSN was 100μg/mL. After treatment, the lower chamber was taken out, the medium in the lower chamber was washed with PBS, and the crystal violet dye was stained for 10min. The crystal violet on the surface was washed with deionized water. The cells in the Transwell lower chamber were photographed under a microscope and the number of cells was calculated to reflect the migration ability of the cells.

**Wounding assay:** The cell density was adjusted to 2.5×10^5^ cells /mL using a blood counting plate, and the cells were inoculated in a six-well plate (2 mL per well) to ensure that the cells could be overgrown on the next day. The cells were cultured in a 5 % CO_2_ incubator at 37 ℃ for 24 h, and divided into 6 groups: (1) Control, (2) RT, (3) Hemin-MSN, (4) GOD@Hemin-MSN, (5) Hemin-MSN+RT, (6) GOD@Hemin-MSN+RT. Among them, the radiotherapy dose was 6 Gy, and the equivalent concentration of MSN was 100 μg/mL. We then use a 200 μL pipette tip to scratch the horizontal line perpendicular to the back of the 6-well plate, 3 lines for each hole. The cells were then cleaned 2-3 times with PBS to remove the scratched cells and fresh 1640 medium was added. The samples were sampled and photographed under fluorescence microscope.

**Animal models：**4 to 5-week-old female BALB/c mice (purchased from Vital River Company, Beijing, China) were subcutaneously injected with 100 μL A549 cell suspension (1×10^7^ cells/mL) on the lateral side of the right leg to establish tumor model. When tumor size reached approximately 200 mm^3^, the mice were divided randomly into 6 groups (each group included 5 mice): (1) Control, (2) RT, (3) Hemin-MSN, (4) GOD@Hemin-MSN, (5) Hemin-MSN+RT, (6) GOD@Hemin-MSN+RT. Among them, the radiotherapy dose was 6 Gy, and an equivalent dose of 0.5 mg/kg mouse body weight MSN was used. Radiotherapy was performed 12h after intravenous injection. Tumor length and width were measured with calipers every 3 days to obtain changes in tumor volume and to record changes in body weight. All procedures have been approved by the guidelines for animal care institutions of Wuhan university and national health institutions.

**Immunofluorescence staining sections:** After 16 days of treatment, the mice were euthanized, and the main organs, including the heart, liver, spleen, lung and kidney, and the tumors, were fixed with 4 % formaldehyde and embedded with paraffin. Dihydroethidium (DHE) was used to stain the tumor sections and fluorescence microscope was used to analyze the ROS in the tumor. The main organs and tumors were treated with TUNEL and Ki-67 staining, and observed with light microscopy for histological analysis.

**Pharmacokinetics curve:** A549-tumor-bearing BALB/c mice (n = 3) received an intravenous injection of 100μL PBS containing GOD@Hemin-MSN (with equivalent MSNs dose of 100 μg/mL). At various time points after the injection (i.e., 0, 4, 8, 12, 16, 20 and 24 h), 20 μL blood plasma was collected from the tail veins, and then centrifugation at 10000 rpm for 10 min. Finally, the supernatants were collected and Si concentration was quantitatively analyzed by ICP AES.

**Biodistribution study of GOD@Hemin-MSN:** A549 tumor bearing BALB/c mice (n = 3) received an intravenous (i.v.) injection of 100 μL PBS containing GOD@Hemin-MSN (with equivalent MSNs dose of 100 μg/mL). All of the mice were euthanized at different time points (1, 12, 24 and 72h), and then we collected their major organs (heart, liver, spleen, lung and kidney) and tumors to determine the biological distribution of the particles. As mentioned above, the Si content was measured using ICP AES.

**HE staining:** At day 16 of the treatment, the mice were sacrificed. The tumor and the main organs including hearts, livers, spleens, lungs and kidneys were harvest, fixed in 4% formaldehyde and embedded in paraffin. The paraffin was sliced at stained with hematoxylin and eosin (H&E).

**Biochemical blood analysis：**When the tumor volume reached 80-100 mm^3^, the untreated group was used as the control group, and the tumor-bearing mice were intravenously injected GOD@Hemin-MSN (an equivalent dose of 0.5 mg/kg mouse body weight MSN), and blood was collected from the orbit of mice at 0.5, 2, 4, 8, 12 and 24 h, respectively.

**Statistical Analysis:** Data were analyzed by one-way analysis of variance (ANOVA) statistical analysis with Sidak’s multiple comparisons test (Graphpad Prism 5.01 software) to determine the differences between groups. A significant difference was judged by P < 0.05.


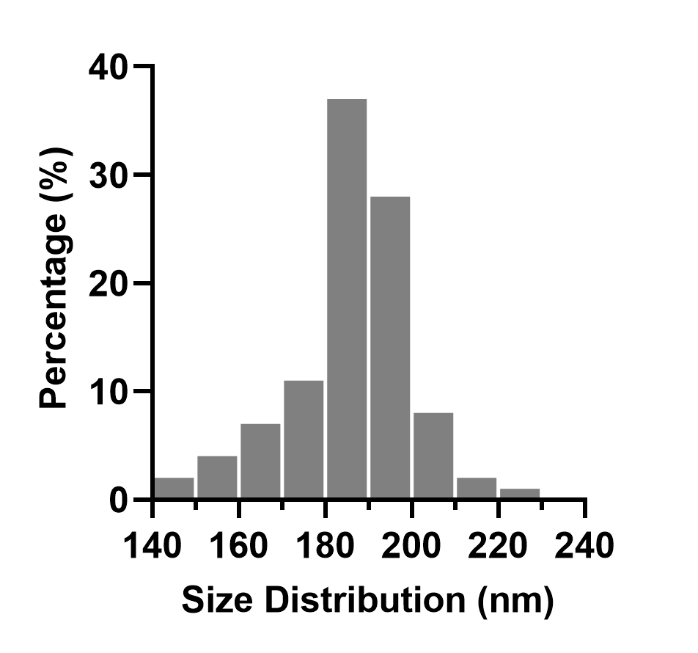


**Figure S1**. Statistical analysis of size of GOD@Hemin-MSN


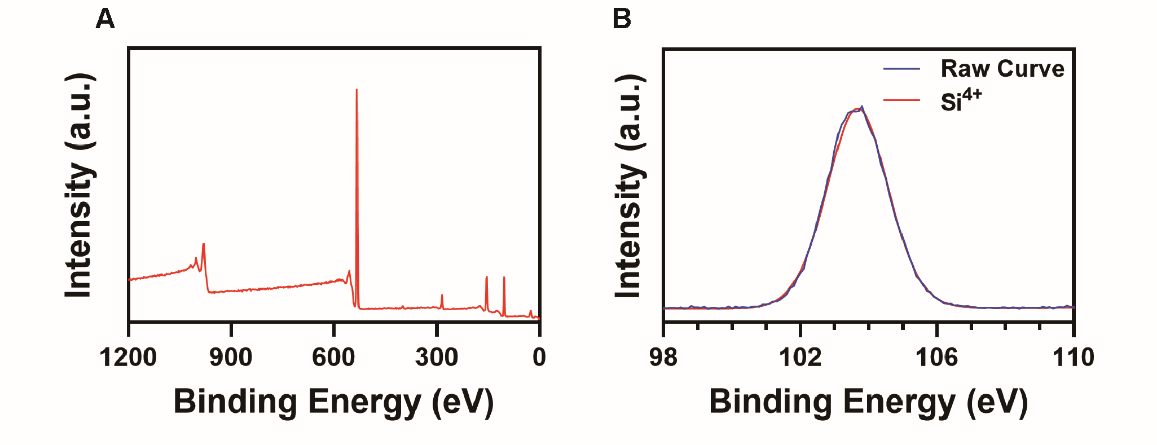


**Figure S2**. XPS spectra of (A) MSN. (B) Si 2p orbit.


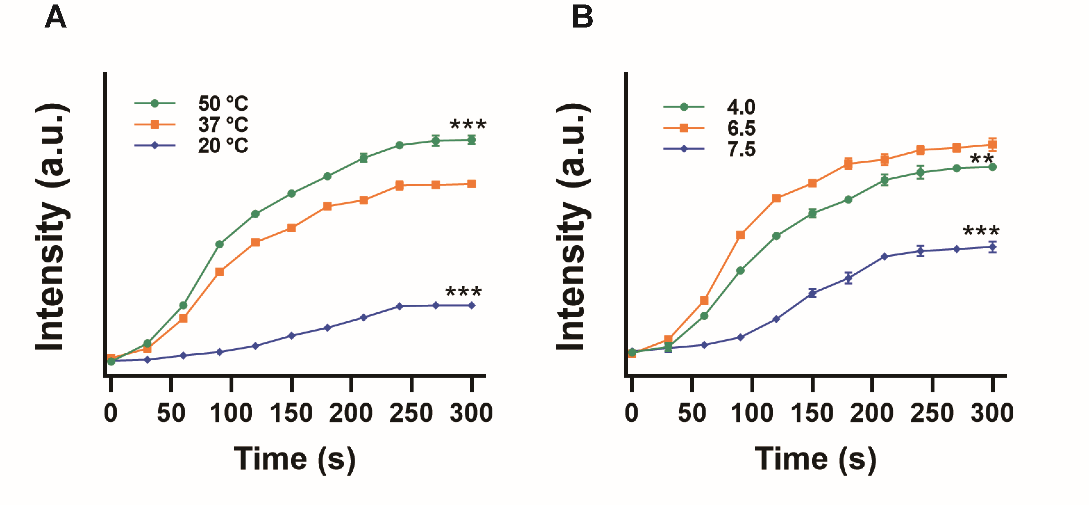


**Figure S3.** Time-dependence absorbance at 652 nm changes of TMB solution with GOD@Hemin-MSN+glucose at different (A) temperature and (B) pH solutions. Data are presented as mean ± SD by one-way ANOVA with Tukey’s multiple comparison tests. ***p < 0.001; **p < 0.01.


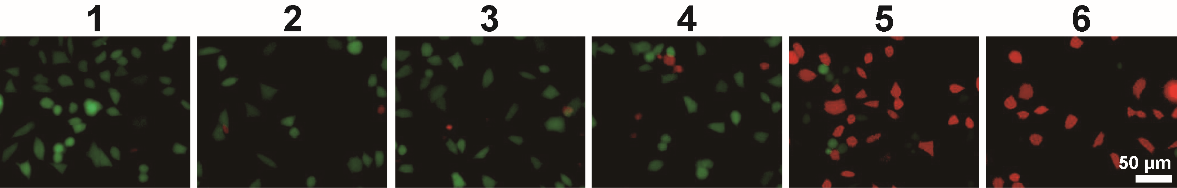


**Figure S4**. CLSM images of live/dead staining in 6 groups. (1. Control, 2. RT, 3. Hemin-MSN, 4. GOD@Hemin-MSN, 5. Hemin-MSN+RT, 6. GOD@Hemin-MSN+RT).


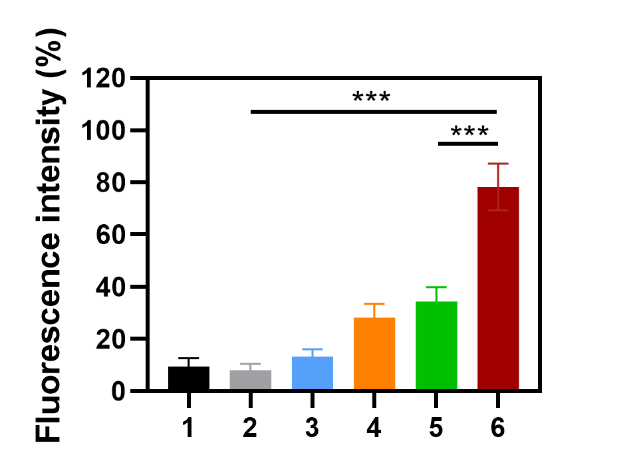


**Figure S5**. Fluorescence intensity of γ -H_2_AX. (1. Control, 2. RT, 3. Hemin-MSN, 4. GOD@Hemin-MSN, 5. Hemin-MSN+RT, 6. GOD@Hemin-MSN+RT). Data are presented as mean ± SD by one-way ANOVA with Tukey’s multiple comparison tests. ***p < 0.001; **p < 0.01 or *p < 0.05. Data are presented as mean ± SD by one-way ANOVA with Tukey’s multiple comparison tests. ***p < 0.001; **p < 0.01 or *p < 0.05.


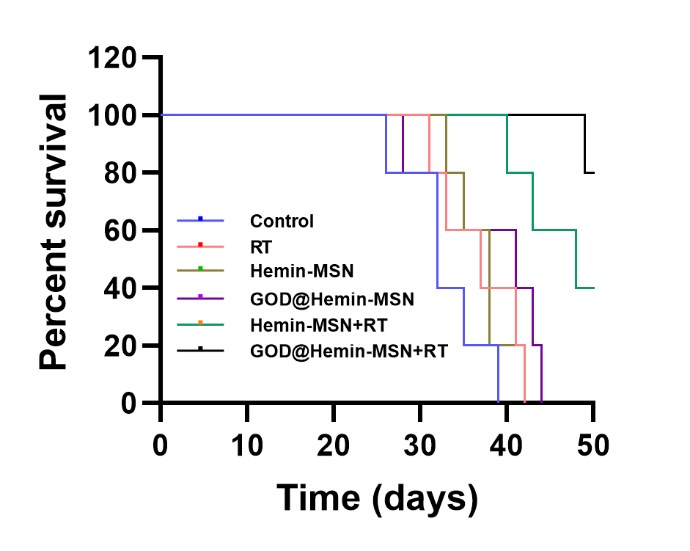


**Figure S6**. Survival curve in various treatments groups.
